# A Configurable Multi-Modal Clinical Time-Series Platform for Predictive Modeling in Cardiology

**DOI:** 10.3390/healthcare14203535

**Published:** 2026-10-20

**Authors:** Gabriel-Mihail Danciu, Stefan Cristian Jarcau, Septimiu Cosmin Nechifor, Andreia-Olga Zaharia, Lefteris Koumakis, Miltiadis Kokkonidis, Nikolaos Vasileiou, Vasiliki Danilatou

**Affiliations:** 1 Faculty of Electrical Engineering and Computer Science, Transilvania University of Braşov (UNITBV), 500024 Braşov, Romania; andreia.zaharia@student.unitbv.ro; 2 Siemens SRL, 062204 Braşov, Romania; 3 SPHYNX HELLAS SA, 166 71 Athens, Greece; 4 Aegis IT Research GmbH, 38106 Braunschweig, Germany; 5 Biomedical Engineering Laboratory, National Technical University of Athens, 15772 Athens, Greece; nvasileiou@biomed.ntua.gr; 6 Eunomia Ltd., D6W FN23 Dublin, Ireland; vicky@eunomia.ltd; 7 Department of Medicine, European University Cyprus, Nicosia 2404, Cyprus; v.danilatou@euc.ac.cy

**Keywords:** multimodal machine learning, clinical time-series prediction, HL7 FHIR, heart failure, remote patient monitoring, explainable artificial intelligence, wearable devices, MLOps

## Abstract

Background/Objective: Predictive models for cardiovascular disease rely predominantly on electronic health records (EHRs), whereas wearable devices, home monitoring, and patient-reported outcomes offer new opportunities for multimodal risk prediction; however, integrating these heterogeneous sources into a reproducible, clinically usable machine learning workflow remains challenging. This study aimed to develop an end-to-end architecture for explainable multimodal time-series prediction in patients with heart failure (HF), left ventricular assist device (LVAD) recipients, and heart transplant (HT) recipients. Methods: The modular architecture harmonizes Health Level Seven Fast Healthcare Interoperability Resources (HL7 FHIR) and non-FHIR telemonitoring data into a unified event log, which leakage-aware temporal preprocessing converts into patient-day feature matrices. Configurable adverse-event tasks (HF hospitalization, cardiac decompensation, transplant rejection, and mortality) are modeled using gradient-boosted trees or temporal neural networks, with optional probability calibration. Explanations combine SHapley Additive exPlanations (SHAP) for tabular models with temporal-perturbation attribution and integrated gradients for sequence models. The pipeline is declaratively configured, tracked with MLflow, and deployable via command-line and web interfaces. It was developed alongside the prospective multicenter RETENTION trial of a telemonitoring-based care pathway and was subsequently applied to data collected within the trial. Results: Applied to the RETENTION cohort, the framework executed the complete workflow, from heterogeneous source data to per-patient-day probability estimates for each prediction task, with feature- and time-level explanations presented as clinician-facing narratives. The cohort served to demonstrate the architecture, not to establish clinical predictive performance or prospective clinical validation. Conclusions: This study provides a reproducible, end-to-end architecture that unifies data harmonization, temporal processing, machine learning orchestration, explainability, and deployment for multimodal clinical prediction. Its modular, disease-agnostic design supports adaptation to other diseases, settings, and data sources, laying a foundation for prospective validation and clinical translation.

## 1. Introduction

Heart failure (HF) is a leading cause of hospitalization and death worldwide, with a global prevalence exceeding 56 million cases and an incidence that continues to rise as the population ages and survival after acute cardiovascular events improves [1]. For patients who progress to advanced HF, a left ventricular assist device (LVAD) or heart transplantation (HT) remains the only definitive treatment option; yet both carry substantial post-intervention risks: contemporary LVAD registries report a five-year survival of only 60% [2], and heart transplant recipients require lifelong surveillance for rejection, infection, and allograft vasculopathy [3]. Early detection of clinical deterioration in these populations (HF decompensation, LVAD-related infection, and transplant rejection) could enable timely intervention and avert preventable hospitalizations and deaths. However, routine care relies on scheduled visits, laboratory draws, and biopsies that sample a patient’s trajectory only at sparse, discrete intervals, leaving the periods between visits clinically unobserved.

Cardiology care increasingly relies on wearable sensors, implantable devices, home-monitoring systems, and tele-monitoring platforms that generate dense temporal data streams, such as heart rate, blood pressure, and oxygen saturation, offering a route to closing this observational gap. In parallel, patients routinely generate heterogeneous clinical data from laboratory tests, imaging, medications, questionnaires, physician encounters, and monitoring, which are collected in different time resolutions, ranging from continuous monitoring to sporadic assessments.

A limited number of clinical trials have explored the integration of remote monitoring and machine learning (ML) approaches in cardiovascular care, particularly in patients with HF. For example, the LINK-HF study demonstrated that continuous remote monitoring using a wearable multi-sensor platform combined with ML algorithms could predict impending hospitalizations in ambulatory HF patients [4]. The ME-TIME study developed a prospective framework for evaluating data-efficient ML approaches, including self-supervised and transfer learning methods, for the detection of atrial fibrillation using multimodal wearable physiological time-series data [5]. More recently, wearable-derived signals have been shown to anticipate HF decompensation ahead of overt symptoms [6], transformer architectures have been applied to longitudinal electronic health record (EHR) data to model patient outcomes with post-hoc explainability [7], and large multimodal foundation models that jointly ingest EHR and imaging data have been proposed as general-purpose clinical decision-support tools [8].

The increasing diversity of multimodal and longitudinal clinical data presents significant engineering challenges related to interoperability, temporal harmonization, reproducibility, and deployment of ML systems. Addressing these challenges requires software architectures capable of integrating heterogeneous data sources while supporting standardized data processing, configurable model development, explainability, and reproducible evaluation workflows. Supporting multimodal clinical prediction within such a reusable framework requires four key capabilities:

1. A canonical temporal representation that hides source heterogeneity;
2. Robust handling of missingness without discarding sparse but clinically informative measurements;
3. a flexible framework supporting multiple prediction tasks and ML model families that jointly capture the static clinical profile and the temporal dynamics of the signals, and;
4. Clinically oriented explanations that support interpretation and decision-making.

Although numerous studies have proposed ML models for cardiovascular prediction, relatively little attention has been given to the underlying software architecture required to support reproducible multimodal data integration, standardized temporal representations, explainability, experiment management, and deployment. The present work addresses this gap by describing the design and implementation of a configurable, end-to-end software architecture for multimodal clinical time-series prediction, extending previous FHIR-based approaches for electronic health records (EHRs) [9] to support heterogeneous clinical and non-clinical data sources.

The primary objective of this work is to design and implement a configurable software architecture for multimodal clinical time-series prediction that provides a standardized framework for integrating heterogeneous longitudinal data, temporal preprocessing, explainability, and reproducible model development. The RETENTION cohort is used as a demonstration use case to illustrate the capabilities of the proposed framework.

The contribution of this work spans four complementary aspects. Methodologically, the framework provides a standardized approach for multimodal temporal representation and configurable prediction-task definition. From an engineering perspective, it integrates heterogeneous clinical and non-clinical data, explainability, and deployment within a unified architecture. From a software perspective, it delivers a modular, configurable, and reproducible implementation suitable for extension and reuse. Clinically, it provides a disease-agnostic platform demonstrated using the RETENTION cohort that can support the future development and evaluation of explainable clinical prediction models. More specifically, the main contributions of this work are as follows:

- **Unified multimodal representation.** A single long-format event log that harmonizes standardized HL7 FHIR clinical resources with non-FHIR remote-monitoring, wearable, and questionnaire data streams behind a common schema.
- **Leakage-aware temporal representation.** A consent-relative time axis with configurable aggregation, missingness-aware imputation, and an explicit mask channel that preserves sparse but clinically informative measurements.
- **Configurable prediction and model zoo:** A declarative prediction-task abstraction spanning binary, time-to-event, count, and multi-class endpoints, served by a unified factory of tabular and temporal models under a common evaluation harness with patient-level cross-validation and MLflow-tracked artifacts.
- **Clinically oriented explainability.** A four-layer stack that translates SHAP, temporal-perturbation, and integrated-gradient attributions into clinician-facing narratives, culminating in an LLM synthesis agent that aggregates these attributions with the prediction and the patient’s history into a single case report.
- **Deployment-ready infrastructure.** An MLflow-tracked, declaratively configured pipeline exposed through both a command-line interface and an interactive operator UI, demonstrated on the prospective RETENTION cohort of advanced HF, LVAD, and heart transplant patients.

The remainder of the manuscript is organized as follows. Section 2 reviews related work and highlights existing gaps in multimodal predictive modeling. Section 3 presents the RETENTION cohort and the conceptual architecture of the pipeline. Section 4, Section 5 and Section 6 describe the data ingestion and harmonization pipeline, the unified event log and patient-day representation, and methods for temporal preprocessing, data completeness assessment and signal reconstruction. Section 7 and Section 8 describe the configurable prediction framework, including prediction-task definitions, supported ML model families, training orchestration, and the validation infrastructure. Section 9 presents the explainability layer, whereas Section 10 and Section 11 describe the graphical operator interface and configuration infrastructure. Finally, Section 12 discusses the key engineering decisions, methodological limitations, and future development directions of the proposed platform, and Section 13 and Section 14 summarize the work and its availability.

## 2. Related Work

Several end-to-end frameworks for clinical AI exist: Rajkomar et al. [9] proposed a scalable FHIR-based deep-learning architecture for EHR prediction, the Holistic AI in Medicine (HAIM) framework demonstrated multimodal integration across clinical modalities [10], Clairvoyance [11] provided a medical time-series pipeline, and PyHealth [12] offers an open-source clinical deep-learning toolkit with more than thirty predictive models. These typically target hospital EHR data, research-oriented modeling, or MLOps deployment separately, and none address advanced HF, LVAD, and HT populations specifically. Moshawrab et al. [13] catalog the corresponding barriers to multimodal ML in cardiovascular prediction—heterogeneous source harmonization, the lack of standardized representations, limited wearable/IoT integration, poor handling of irregular longitudinal data, and the absence of interpretable, deployable frameworks—which the RETENTION pipeline addresses through a unified event-based representation of standardized HL7 FHIR and non-FHIR streams with explicit temporal alignment, missing-data reconstruction, multimodal fusion, explainability, and MLflow-tracked deployment.

The individual techniques used here—SHAP explainability [14], HL7 FHIR integration [15,16], Transformer architectures [17], MLflow tracking [18], and Streamlit deployment—are well established and are not claimed as contributions in themselves. The novelty is instead *integrative* and spans four axes: *methodological* (a consent-relative, leakage-aware temporal representation with an explicit missingness mask, and a multi-layer explainability stack producing a common clinical narrative); *engineering-integration* (to our knowledge, among the first to combine standardized FHIR and non-FHIR wearable/remote-monitoring streams, a multi-family model zoo, and MLOps in one configurable pipeline, unlike HAIM, Clairvoyance, or PyHealth); *software-implementation* (a declaratively configured, MLflow-tracked, dual-interface CLI/UI system rather than a research codebase); and *clinical* (a prospective, multicenter cohort spanning HF, LVAD, and HT, paired with an LLM narrative-generation service [19]).

Table 1 positions the present framework against these systems along the four capabilities set out in Section 1. Two observations follow. First, the individual capabilities are, without exception, available somewhere in the existing literature: FHIR-based representation was established by Rajkomar et al. [9] and is now also supported by PyHealth 2.0 through a generic loader for FHIR exports; multimodal fusion and Shapley-based attribution are central to HAIM; configurable problem definitions, imputation, and instance-wise interpretation are provided by Clairvoyance; and a broad model zoo with a unified trainer is the core of PyHealth. We therefore claim none of these individually. Second, the combination is nonetheless unmet: no compared system ingests from a live authenticated FHIR endpoint, none fuses standardized clinical resources with patient-generated remote-monitoring streams into a single longitudinal representation with an explicit observed-versus-imputed mask, none couples that representation to a model registry and a non-programmer operator interface, and none targets the advanced heart-failure, LVAD, and transplant population. The contribution of this work is that combination, and the table is offered as a scope comparison, since each compared system was designed against different requirements and several remain stronger than ours on axes we do not address, notably AutoML in Clairvoyance and breadth of model implementations and dataset loaders in PyHealth.

## 3. Patient Cohort Used to Demonstrate the Proposed Software Framework

The proposed software framework was developed and demonstrated using data from the RETENTION randomized clinical trial (NCT07008729), a prospective multicenter European study investigating multisensory remote monitoring in advanced HF, LVAD, and heart transplant (HT) patients. The study combines longitudinal clinical data with continuous streams from wearable devices, home-monitoring systems, questionnaires, and environmental sensors through a dedicated digital health platform, providing a comprehensive multimodal dataset for AI-driven predictive modeling and personalized cardiovascular care [20]. This multimodal environment provides a representative use case for demonstrating the proposed software architecture.

The RETENTION trial was conducted in accordance with the Declaration of Helsinki, Good Clinical Practice [21], and applicable EU and national legislation [22], with ethical approval obtained from the competent authorities of all participating institutions prior to recruitment. Participation was voluntary, with written informed consent obtained from each patient before any study procedure. Personal and health data were processed under a framework compliant with the EU General Data Protection Regulation (GDPR) [23], incorporating pseudonymization, encryption, and controlled access, with data anonymized prior to model training. In line with the European Commission’s Trustworthy AI guidelines, the project applied the Assessment List for Trustworthy Artificial Intelligence (ALTAI) [24] as an iterative self-assessment tool across model design, development, and validation.

### 3.1. RETENTION Study

**Figure 1:**
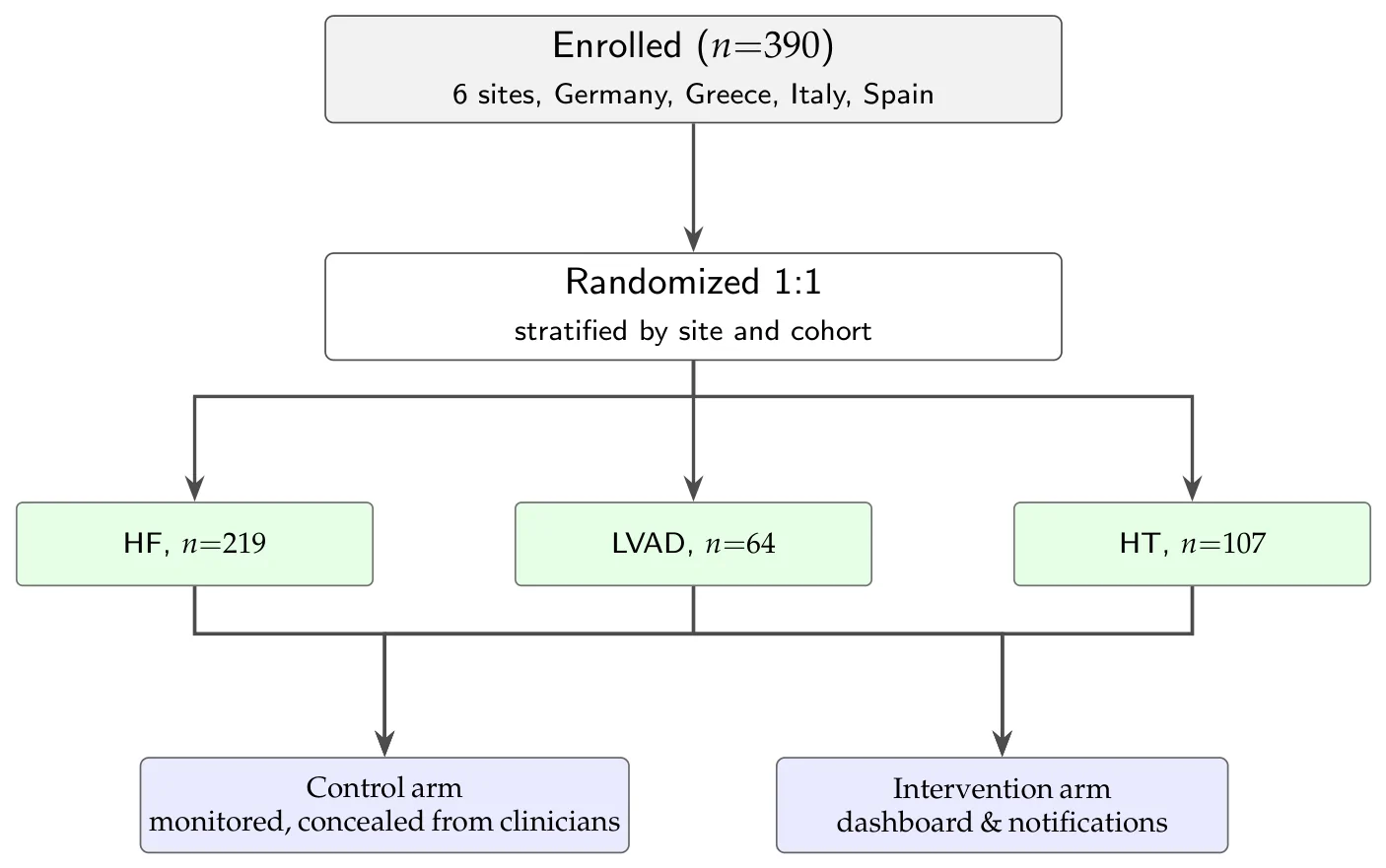
Recruitment and randomization flow. Patients enrolled at six European sites were randomized 1:1, stratified by site and clinical cohort, into HF, LVAD, and HT subgroups and, within each subgroup, into a control or an intervention arm. The pipeline described in this paper is trained on data pooled across sites and arms; it does not model or condition on the randomization arm.

RETENTION is a prospective, multicenter, randomized, controlled trial conducted at six European hospitals in Germany, Greece, Italy, and Spain, enrolling 390 patients aged 18 to 75 years across three subgroup-specific inclusion criteria: symptomatic HF (NYHA class II or III, left ventricular ejection fraction ≤40%, and a cardiovascular hospitalization or emergency visit requiring intravenous diuretics within the previous 12 months, not listed for advanced therapies at enrollment; n=219); LVAD support, implanted 90 days to 48 months before randomization, either as destination therapy or as a bridge to transplantation (n=64); and HT recipients 30 days to 36 months after transplantation at randomization (n=107). Eligible participants were randomized 1:1 to a control or an intervention arm, stratified by center and clinical cohort. Both arms received the same monitoring devices; the intervention arm’s clinicians additionally had access to a web-based dashboard and protocol-based notifications, while the control arm’s transmitted data were stored but concealed from the treating clinical team. Study visits were scheduled at baseline and every 4 months, which is the origin of the periodic-measurement cadence referenced in Section 6. Figure 1 summarizes this recruitment and randomization flow; the pipeline described in the remainder of this paper operates on the data these patients generated.

### 3.2. Cohort Snapshot

**Figure 2:**
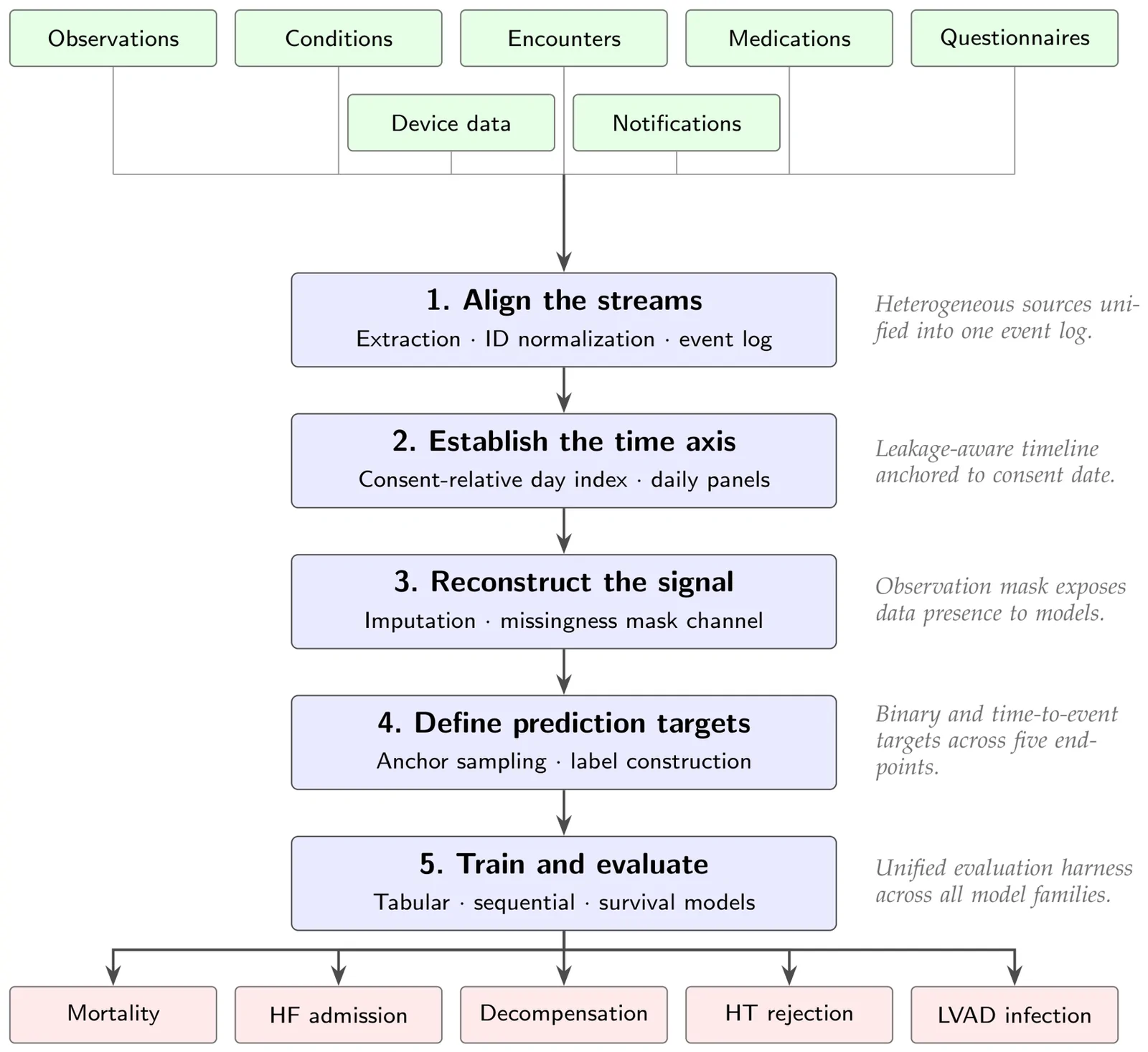
Five-stage conceptual pipeline. Clinical streams (FHIR and non-FHIR) are aligned into a long-format event log, projected to daily panels on a consent-relative time axis, reconstructed through completeness filtering and imputation, labeled via the prediction-task abstraction, and fed to a mixed tabular/sequence model zoo. Adverse outcomes span mortality, HF admission, HF decompensation, HT rejection, and LVAD-related infections.

The RETENTION trial was initiated in 2019 as a prospective, multicenter, randomized clinical trial evaluating a telemonitoring-based care pathway. Recruitment and study progression were substantially disrupted by the COVID-19 pandemic, resulting in slower-than-anticipated enrollment and an extension of the study until September 2026. The software infrastructure was developed in parallel with the conduct of the clinical trial, using data collected during the study. Thus, the initial demonstration of the framework, whose five-stage conceptual pipeline is outlined in Figure 2, depends on retrospectively used data. At the time of writing, the cohort included 390 patients and more than 162,000 patient-day observations derived from heterogeneous clinical and remote-monitoring sources. The temporal dataset comprises 145 feature variables collected over an initial observation window (2019–2021). Clinical outcome labels used in the present framework demonstration were derived from 1239 recorded encounters and have not yet undergone formal clinical adjudication. A clinician-led adjudication process is planned to establish the definitive outcome definitions for subsequent benchmarking and validation studies. In the present demonstration, unscheduled encounters are used as a proxy for HF-related admissions. An unscheduled encounter is a surrogate rather than an adjudicated HF hospitalization: it may capture urgent contacts unrelated to HF and may miss admissions recorded in other systems. These labels are therefore used to exercise the framework, not as validated clinical endpoints, and the planned clinician-led adjudication will replace them for subsequent benchmarking and validation.

## 4. Data Sources and Stream Alignment

In routine clinical care, patient information is distributed across multiple systems and collected at different time points and frequencies. This section describes how the proposed framework harmonizes these diverse data sources into a standardized longitudinal dataset suitable for downstream ML predictive analytics. The process comprises three stages: data ingestion from heterogeneous sources, stream alignment into a common representation, and construction of a unified longitudinal event log for subsequent analysis.

### 4.1. Ingestion

Two acquisition layers sit in front of the pipeline. The first is an OAuth2-authenticated client for a configurable HL7 FHIR [15,16] R4 endpoint: a token manager obtains, caches, and refreshes bearer tokens against the project’s OAuth2 realm, transparently retrying on HTTP 401; a paginated FHIR reader follows both Bundle.link[next] and offset-based idioms, streaming each page to NDJSON so downloads survive interruption before being consolidated into a single JSON cache per resource (configurable page size, _count=1000 by default); and a per-patient fetch driver retrieves Patient, Observation, Condition, Encounter, MedicationStatement, and QuestionnaireResponse resources. The second layer wraps a proprietary non-FHIR REST API exposing device measurements and notification events, reusing the same token manager and adding a file-based TTL cache that enables offline replay. All endpoint locations, realms, and credentials are supplied through configuration and environment variables and are not hard-coded, allowing the ingestion layer to operate with any conformant FHIR R4 server.

### 4.2. FHIR Extraction

Raw FHIR bundles are flattened into tabular form by resource-specific extractors that enforce two invariants: (i) one row per clinically atomic event (a blood-pressure observation with systolic and diastolic components emits two rows, one per component) and (ii) deduplication by resource identifier across overlapping cache files. The output is five CSV files sharing a common spine: patient identifier, timestamp, code, display label, value, value unit, and domain-specific columns.

### 4.3. Patient-ID Normalization

Sources disagree on the patient-identifier convention, numeric in one export and prefixed in another, so a single normalization step coerces every source to a canonical form before any downstream join. This is why all subsequent merges are trivial: every loader funnels through the same normalization helper.

## 5. Clinical Event Integration and Patient-Day Representation

Clinical events are recorded at irregular time points and in different formats. This section describes how the proposed framework integrates these events into a standardized patient-day representation through event integration, temporal alignment, and daily aggregation.

### 5.1. The Unified Event Log

The central abstraction is a long-format event table produced by concatenating all per-source CSV files. Two data dictionaries are merged into a single code-to-display mapping so that every event receives a human-readable variable name. Embedded FHIR Observation component entries are parsed and emitted as additional event rows, preserving the parent category. Table 2 shows the resulting nine-column schema with representative rows from each source.

### 5.2. Consent-Aligned Time Axis

**Figure 3:**
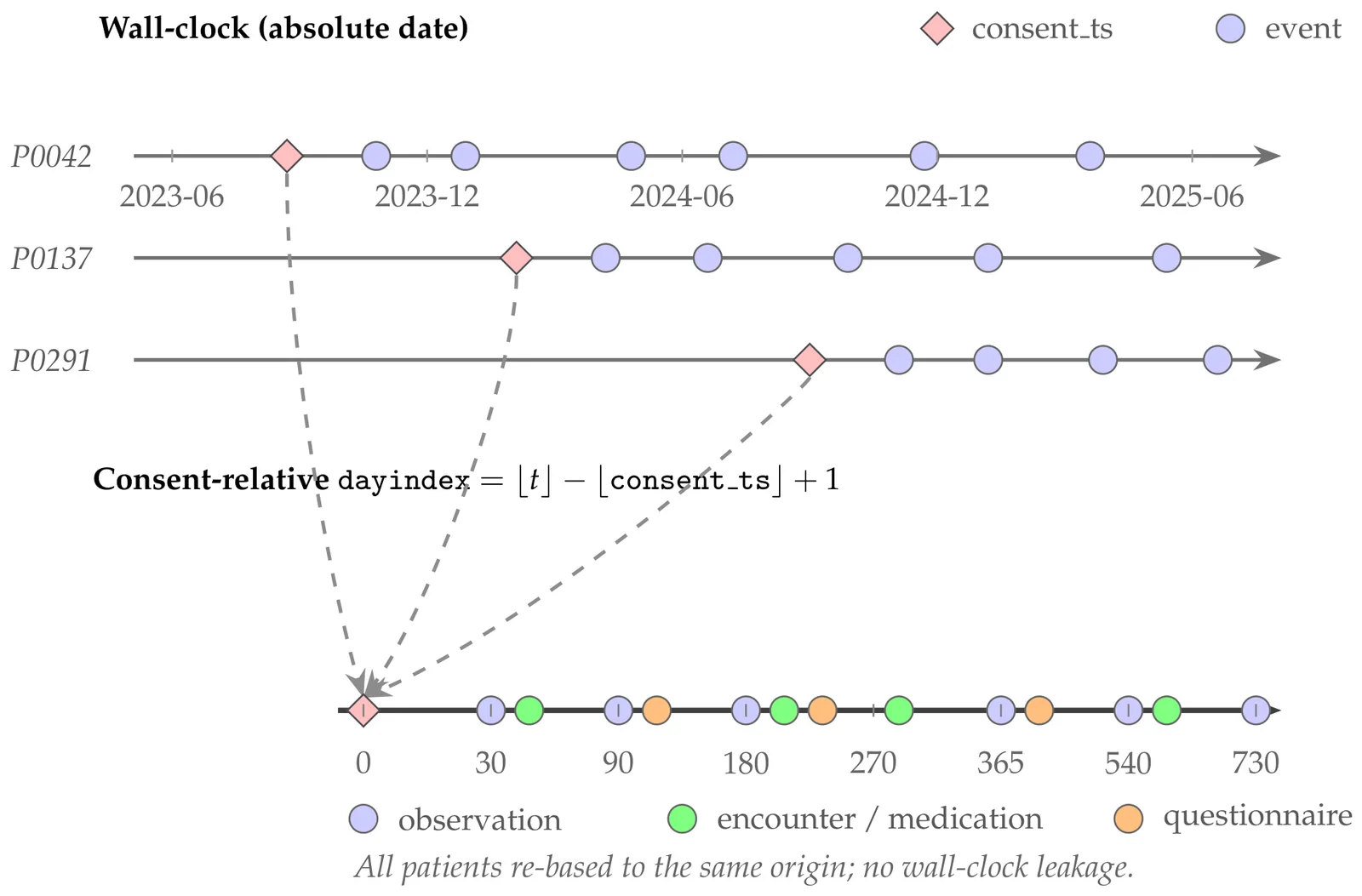
Consent-relative time axis. Three patients enrolled on different calendar dates have their event streams re-based to a common day-index origin, allowing the model to use time since consent without relying on absolute calendar dates. Calendar-derived signals (e.g., seasonality) are recoverable as static features from the consent timestamp.

Every patient has a consent timestamp recorded at enrollment. The event log stores a day index defined as

$$\mathrm{dayindex}=\left\lfloor \mathrm{timestamp}\right\rfloor -\left\lfloor \mathrm{consent}\_\mathrm{ts}\right\rfloor +1,$$

which re-bases every patient to the same origin. All subsequent windowing, splitting, and visualization are done on the day index instead of absolute dates: the model never sees wall-clock time, only time-since-consent, which prevents leakage from cohort calendar effects. Figure 3 illustrates the collapse.

### 5.3. Daily Panels and the Wide Matrix

The event log is projected to a dense $\left[N_{\mathrm{pat}},T,F\right]$ tensor by an aggregation pipeline. The aggregation step is pluggable: an identity strategy keeps raw daily granularity; weekly and four-month strategies re-bin for studies where the signal is slow-changing. Within each bin, continuous variables are reduced by the mean and categorical variables by the last value, with counts preserved for rare events. Variables are classified by frequency (daily, continuous-daily, periodic, sporadic, and annual), and the aggregation applies a strategy appropriate to each class.

## 6. Missing Data Handling and Signal Reconstruction

Clinical data are inherently sparse and irregularly sampled. This section describes the two components of the framework that preserve information on data completeness while reconstructing longitudinal patient signals for downstream analysis.

### 6.1. Data Completeness Assessment

A completeness analyzer computes per-patient, per-variable, and per-cohort coverage statistics, renders them as heatmaps stratified by clinical site, patient category, and treatment group, and exposes milestone-aware drill-downs (e.g., did the patient have a measured 6-min walk at the three-month milestone?). A variable filter applies a configurable minimum-coverage threshold so that features present in too few patients never reach a model.

Coverage is assessed against each patient’s own observation window, defined as the interval from the patient’s consent date to their last recorded observation with right censoring at withdrawal or death where applicable, instead of using a fixed calendar period. This avoids penalizing patients with shorter follow-up for gaps they could not have filled. Within that window, variables are classified into three frequency classes prior to computing coverage: daily measurements (e.g., heart rate, weight, smartwatch metrics, expected on most days), periodic measurements (e.g., laboratory panels and echocardiography, sampled at coarser, clinically driven intervals such as the roughly four-month study milestones), and discrete events (e.g., hospital admissions, infections, recorded as point-in-time flags and not as continuous signals). Coverage is reported as the fraction of patient-days with directly observed cell-level data and does not rely on an inferred expected-sampling denominator because informal, clinically determined periodic-visit schedules are not followed by design. We report coverage descriptively and do not attempt to determine the reasons for missing data; establishing the underlying missingness mechanism would require modeling the relationship between missingness and both observed and unobserved values.

### 6.2. Signal Reconstruction

A data cleaner catches physiologically impossible values (zero heart rate, zero systolic blood pressure, SpO_2_ outside [50,100]) and substitutes missing values before imputation. Eleven imputation methods are implemented (linear, cubic, Akima, forward/backward fill, mean, median, polynomial, exponentially weighted, Gaussian smoothing), benchmarked head-to-head, and regression-tested. The default is linear interpolation for short gaps in continuous daily variables and forward-fill for categorical flags; this policy is configurable for each variable. Because most gaps arise from mechanical or schedule-driven sparsity, such as irregular visit timing or device connectivity, and are not attributable to clinically motivated non-response, the default imputation strategy favors low-bias, shape-preserving interpolation for densely sampled continuous variables and carry-forward for slowly changing or categorical signals. The explicit mask channel described below is used when imputation confidence is low.

Sequence models also receive a *mask channel* [25] so the model can distinguish between imputed and observed values and account explicitly for their different levels of reliability.

## 7. Prediction Tasks Formulation

This section describes how prediction endpoints are formulated through a configurable task abstraction that supports multiple outcome types and ML model families (model zoo) within a common software architecture.

A PredictionTask abstraction specifies a supervised-learning problem through the following fields: objective (∈ {classification, regression}), data mode (∈ {tabular, sequence}), label kind (∈ {binary-within-horizon, time-to-event, multiclass, count-within, length-of-stay}), target column, label builder, lookahead window, and cohort filter. Built-in cohort filters select either the full cohort or only HF/VAD patients; built-in label builders scan encounters and observations for keywords indicative of the outcome (e.g., emergency, hospital, ICD/SNOMED-coded HF decompensation rules) and assign a positive label within the lookahead window. These are rule-derived, unadjudicated labels. The current task roster, including a general cardiovascular (CV) admission endpoint, is summarized in Table 3.

## 8. Predicting Adverse Events

Following data harmonization, temporal preprocessing, and prediction-task definition, the proposed framework provides a standardized infrastructure for developing ML models for clinically relevant adverse events. This section describes the configurable prediction framework, including the supported model families, training workflow, representation of static and longitudinal patient information, and the validation protocol used to ensure reproducible and leakage-aware model development.

### 8.1. Model Zoo

**Figure 4:**
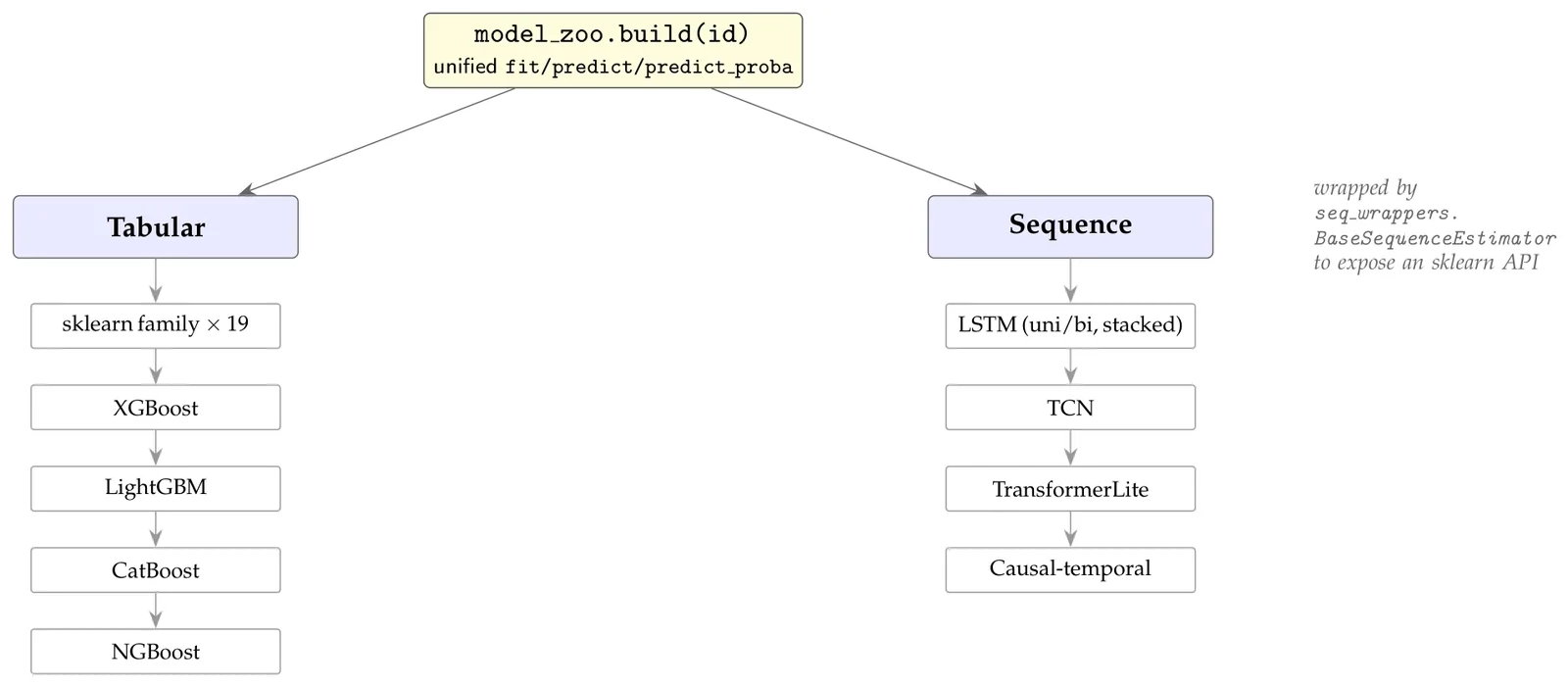
Model-zoo taxonomy. The factory returns a scikit-learn-compatible estimator regardless of whether the underlying implementation is a classical sklearn model, a third-party boosting library, or a PyTorch sequence model.

A uniform model factory exposes more than 25 template identifiers (Figure 4). The tabular branch spans a scikit-learn family alongside gradient-boosted implementations, namely XGBoost [26], LightGBM [27], CatBoost [28], and NGBoost [29], while the sequence branch provides LSTM [30], temporal convolutional [31], and Transformer [17] architectures. All models are wrapped by a universal model wrapper that presents a common build/fit/predict/ predict-probability interface. Sequence models additionally go through a base sequence estimator that hides PyTorch device placement and tensor shapes behind the scikit-learn contract, so the same evaluation harness works for every family.

### 8.2. Training Orchestration

**Figure 5:**
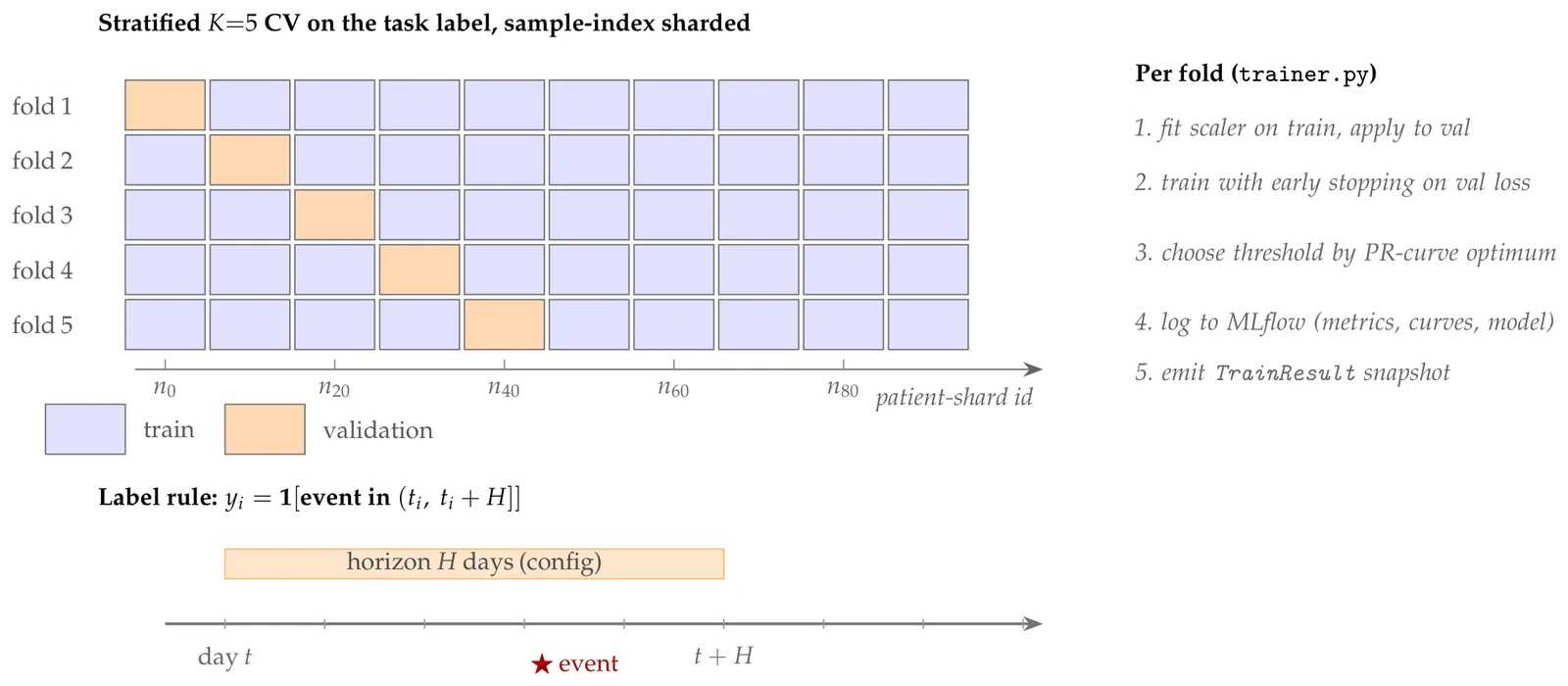
Stratified *K*-fold cross-validation. Each fold fits a scaler on the train partition, trains with early stopping on the validation partition, selects a decision threshold from the precision-recall curve, logs artifacts to MLflow, and emits a result object. The bottom strip illustrates the horizon-window label rule shared by all binary-within-horizon tasks.

The training loop exposes epochs, batch size, learning rate, early-stopping patience, device, loss kind (BCE, focal [32], MSE), focal-loss γ/α, class weighting, and SMOTE oversampling [33] toggle, and gradient-clipping norm. A training result object captures the best-epoch snapshot, per-fold and per-category metrics, predictions, fitted scaler, and the threshold chosen by precision-recall optimization on the validation split. *K*-fold cross-validation is driven by stratification on the task label, with the clinical site used as an additional stratification variable so that every fold and the held-out evaluation bucket contain a balanced representation of patients from each participating clinical site; the scheme is sketched in Figure 5. This design distributes site-level variation across folds without isolating individual sites and should not be read as a leave-one-site-out or other externally validated protocol (Section 12).

MLflow [18] is the system of record for experiments. The logging layer records hyperparameters, loss curves, validation-metric progression, confusion matrices, ROC curves, feature-importance plots, and model artifacts per run. A separate experiment aggregator collects runs across tasks, cohorts, and model combinations for post-hoc comparison; checkpoint persistence is versioned by task identifier and fold.

### 8.3. Static and Sequence Context

Per-patient baseline features, namely age, gender, body mass index (BMI), baseline labs, and comorbidity counts, are extracted and concatenated to the sequence tensor as a broadcast time-invariant channel. This is the cleanest way to provide recurrent models with the static profile without a separate tower; for gradient-boosted trees, the static features simply join the flat feature vector.

### 8.4. Validation Protocol and Leakage Control

#### 8.4.1. Patient-Level Partitioning

All partitioning is performed at the patient level, never at the anchor level. The 82 patients contributing HF-admission anchors are assigned to five folds, stratified by patient-level outcome status so that each fold contains a comparable proportion of patients with at least one positive anchor. Every anchor derived from a given patient is assigned to that patient’s fold, so no patient appears on both sides of any split. Because a patient contributes ≈28 anchors on average, anchor-level splitting would place near-duplicate observations of the same trajectory in both training and test; the patient-level constraint prevents this by design and eliminates the need for post-hoc filtering. For the same reason, all confidence intervals are computed by bootstrap resampling over patients, not over anchors, so that the unit of statistical analysis matches the unit of partitioning.

#### 8.4.2. Temporal Validation

Each anchor is evaluated causally on the consent-relative axis: features are derived exclusively from the observation window preceding the anchor timestamp, and the prediction horizon is strictly disjoint from that lookback window, so no information from the label interval enters the feature representation. The mask channel records observation status as of the anchor time and is never backfilled from later observations. A held-out prospective time window is not feasible at this cohort size without reducing the positive count below the level needed for informative comparison; within-patient temporal causality is enforced as described above, and prospective validation is identified as future work.

#### 8.4.3. Prevention of Data Leakage

All fitted preprocessing is estimated on training folds alone and applied unchanged to validation and test folds: normalization statistics, imputation parameters, missingness encodings, class-reweighting factors, and any feature selection or dimensionality reduction. Static context (age, sex, comorbidity burden) is drawn from the record as of the anchor time. Multiple anchors arising from the same clinical episode remain within a single fold as a consequence of patient-level assignment.

#### 8.4.4. Model Selection

Hyperparameter selection and early stopping use a patient-disjoint validation split drawn from within the four training folds only; the test fold is not consulted during selection and is evaluated once per fold. Selection uses AUPRC, and early stopping is governed by the validation AUPRC under a fixed patience budget applied identically across templates. The per-template hyperparameters and stopping patience are declared through the same YAML configuration surface (Section 11), so that comparisons are not confounded by unequal tuning budgets.

## 9. Explainability

Accurate predictions alone are insufficient for clinical adoption; clinicians also require transparent and interpretable explanations to understand and trust model outputs. This section describes the explainability framework integrated within the proposed architecture, which combines complementary attribution methods with clinician-oriented narratives to support interpretation of model predictions.

**Figure 6:**
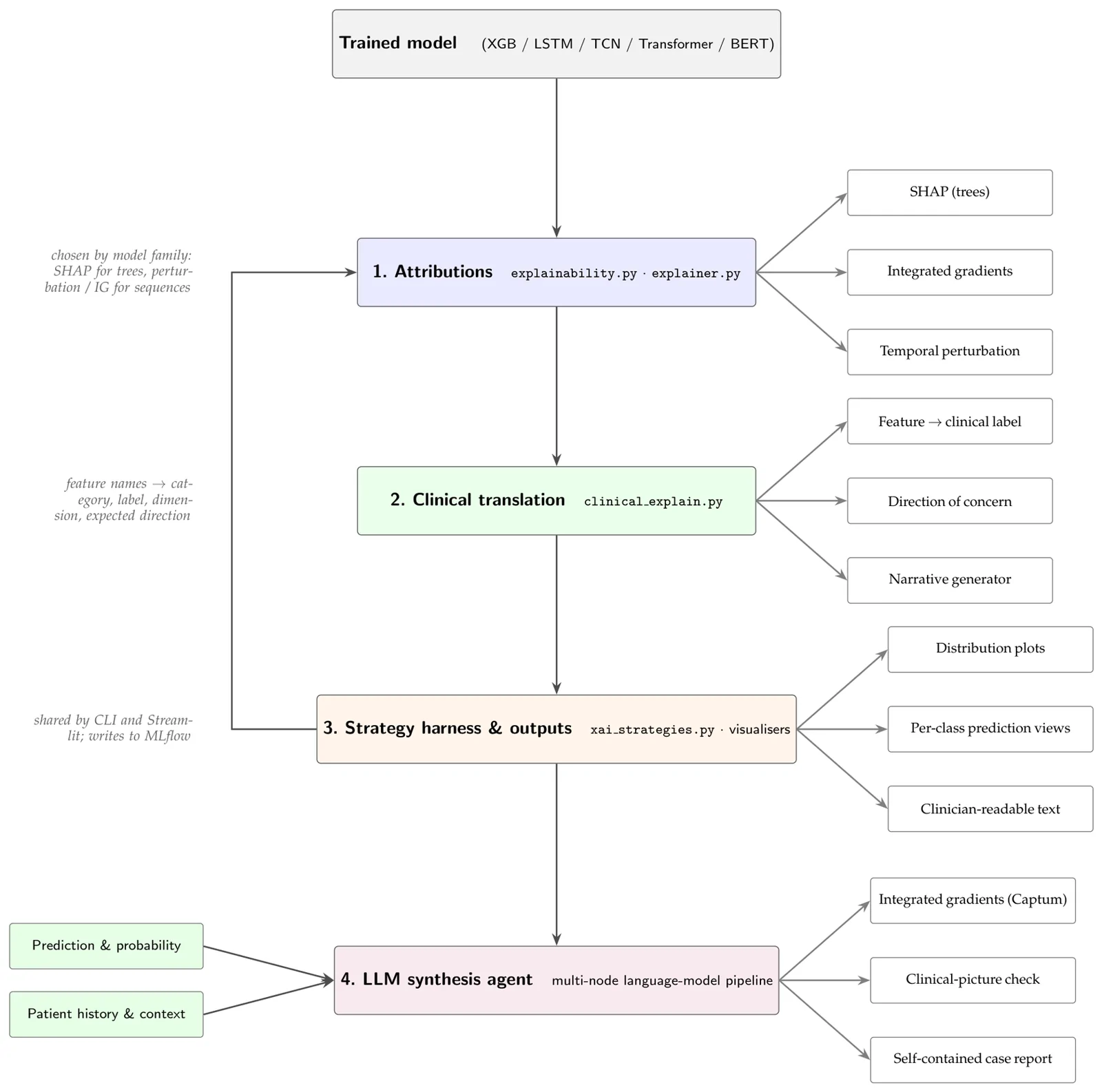
Four-layer explainability stack. Attributions are chosen by model family; the clinical-translation layer converts feature names into categorized descriptors with direction-of-concern metadata; the strategy harness assembles plots and narratives that are reused by both the command-line interface and the interactive UI; the LLM synthesis agent aggregates all three layers, together with the prediction and the patient’s history, into one case report.

Clinical explanations are produced by four cooperating layers (Figure 6), the first three of which run inside the training and evaluation pipeline, and the fourth of which runs at deployment time to synthesize their output, along with the prediction itself and the patient’s history, into a single clinician-facing report.

1. **Global and local attributions.** An explainability engine wraps each trained model and exposes a batched probability-domain prediction function, a temporal-importance scorer, and plot generators. For tree models, attributions are SHAP values [14,34]. For sequence models, *temporal-perturbation attribution* estimates each time step’s contribution by overwriting it with the cohort mean and measuring the resulting change in predicted probability; integrated gradients [35] are supported as an alternative on the LSTM head.
2. **Clinical translation.** A clinical-translation layer maps raw feature names onto their clinical descriptors, namely category, human-readable label, dimension, and expected direction of concern, and emits narrative text such as: “The model’s concern is driven mainly by rising resting heart rate (+12 bpm vs. baseline) and falling SpO2_2 over the last 7 days, partially offset by stable weight.”
3. **Strategy harness.** A strategy harness composes the two layers above into reusable analyses: an input-statistics and prediction-distribution report, feature-distribution and prediction-by-class visualizations, and a 2-D scatter/heatmap of model behavior conditioned on the two most important features.
4. **Experimental LLM synthesis agent.** A deployment-time service takes the outputs of the three layers above, namely the local attributions, the clinical-translation metadata, and the strategy harness’s visual summaries, together with the model’s prediction and probability for one patient and that patient’s recent observation history and clinical context, and drives a multi-step large-language-model pipeline over them. The pipeline ranks the attribution drivers, checks the prediction against the broader clinical picture, flags statistical concerns in the patient’s history, and synthesizes all of this into one self-contained case report, intended as a clinician-facing companion to the templated narrative from layer 2. This layer is an experimental explanatory output; its statements are not verified against the source data, rely on clinician verification, and it is not a decision-support recommendation.

**Figure 7:**
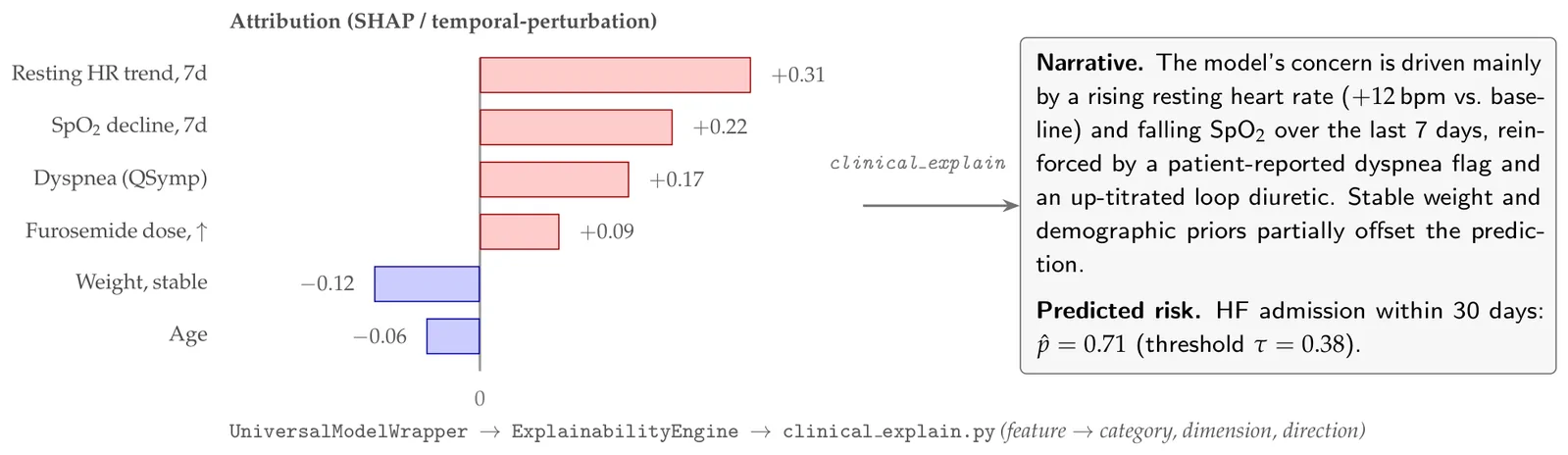
From attribution to narrative. Ranked per-feature contributions (positive values push predicted risk up, negative values offset it) are translated into a short narrative by the clinical-translation layer, using each feature’s category, dimension, and direction-of-concern metadata. The decision threshold τ is selected by precision-recall optimization during training. The horizontal arrow labeled clinical_explain denotes this translation step; the arrows in the bottom strip trace the processing path through the software components and the metadata fields assigned to each feature (category, dimension, direction); the upward arrow next to “Furosemide dose” indicates an increased dose.

Visualization components are thin view layers over the first three engines and are shared between the training scripts and the interactive UI. Figure 7 shows a representative end-to-end example, from raw attributions to the clinician-facing narrative produced by the clinical-translation layer.

### LLM-Generated Case Report

Unlike the fixed-template narratives of layer 2, the fourth layer’s report is generated freely by the language model instead of assembling text from a predefined set of sentence templates, allowing it to identify surface patient-specific caveats, such as flagging implausible or conflicting observations that a template cannot anticipate in advance; this flexibility is also why its output requires the safeguards discussed below. The report is rendered as a single self-contained document embedding the same attribution visualizations produced by layers 1 to 3 alongside the language model’s narrative, and every report carries an explicit notice that its content is AI-generated, may contain unsupported or inaccurate statements, and must be independently verified by a qualified clinician before informing any clinical decision. This approach follows a broader pattern in recent clinical AI work of pairing feature-attribution methods with large language models to translate quantitative model output into clinician-facing prose [19].

## 10. Operator Interface

To improve accessibility for clinicians and researchers, the framework includes a graphical operator interface that allows users to interact with the platform through an intuitive workflow instead of command-line tools. This section presents the main features of the interface and how they support the end-to-end ML pipeline.

**Figure 8:**
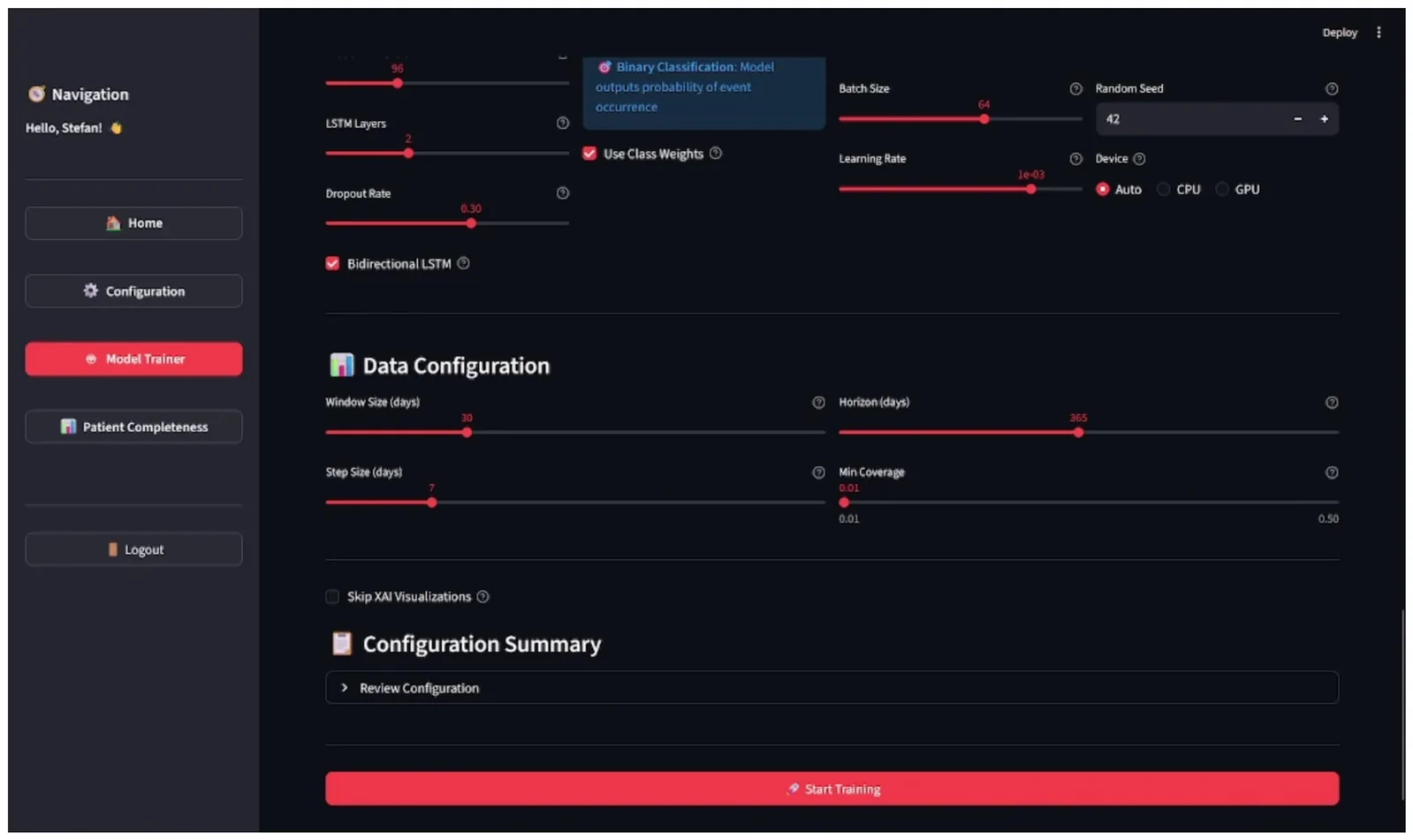
GIC Trainer and Statistics web application. Sidebar navigation on the left switches between Home, Configuration, Model Trainer, and Patient Completeness; the main panel shows the Model Trainer page with trainer hyperparameters, the data configuration block, and the *Start Training* action that dispatches run_pipeline in-process.

The pipeline is fronted by a Streamlit application, the *GIC Trainer and Statistics* interface (Figure 8), which runs on the Global Insights Cloud (GIC) instance alongside the data layer and the MLflow tracking server. It is the single entry point through which a researcher configures the cohort, inspects data quality, trains a model, and hands the resulting artifacts off to the MLflow registry. Importantly, the UI owns no data or modeling logic: every page calls directly into the same pipeline, trainer, completeness, and explainability components that back the command-line interface, which keeps batch and interactive runs bit-identical.

The interface exposes four pages behind a lightweight session/role gate (Researcher, Data Analyst, Clinician, Administrator): a *Home* landing page; a *Configuration* page that edits the pipeline’s single YAML configuration file through five tabs mirroring Section 11; a *Model Trainer* page (Figure 8) that launches run_pipeline from the same TASKS registry used by the CLI, streams live per-epoch status, and renders the resulting metrics and explainability artifacts; and a *Patient Completeness* page that surfaces per-site, per-patient, and per-variable coverage diagnostics against the events-long table. Full page-by-page and tab-by-tab detail is given in Appendix A.

**Figure 9:**
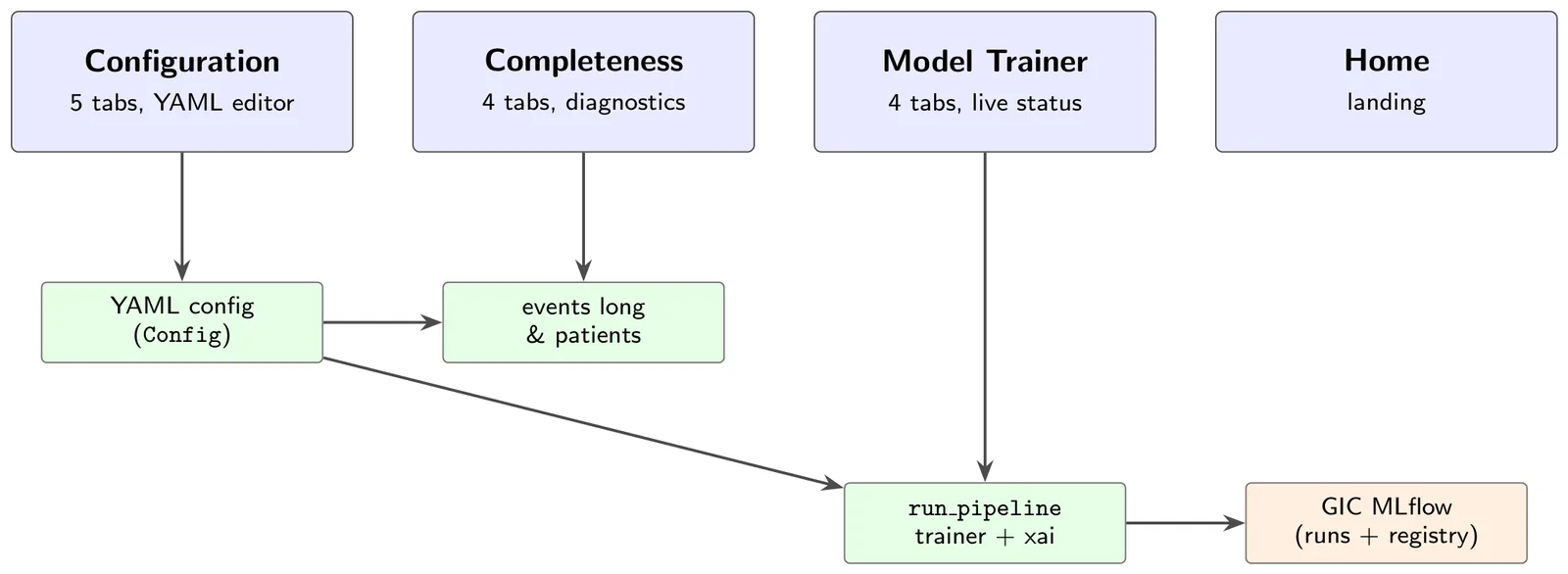
GIC-side operator interface. The configuration page writes the YAML consumed by every other component; the trainer and completeness pages call the same pipeline entry points that back the CLI; trained runs land on the GIC MLflow registry, from which approved versions are exported to hospital deployments.

Figure 9 illustrates how the four pages map onto the rest of the pipeline: the configuration page edits the YAML that drives every component; the completeness page reads from the events long table; the trainer page calls the same run_pipeline entry point that the CLI uses and writes to the GIC-side MLflow registry, from which the deployment time exporter (Section 12) eventually ships models to the hospital instances.

## 11. Configuration Surface

Configurability is a fundamental design principle of the proposed software architecture. This section describes the centralized configuration surface that enables adaptation of the framework to different datasets, prediction tasks, ML models, and deployment environments while maintaining reproducibility.

A single YAML file at the repository root declares everything that should not live in code and is the contract between the CLI, the pipeline modules, and the operator interface. The same file backs the five tabs of the configuration page (Section 10) and is revalidated through the typed Config object whenever it is saved. Its top-level keys cover source and artifact *paths*; *cohort enumerations* (patient category, treatment group, clinical site identifier, recruitment status); the *variable taxonomy* used by the aggregation strategy and completeness reports; *sequence parameters* (horizon, window, step, bin size, minimum coverage, mask channel toggle); *evolution targets* for the better/stable/worse task; *HF endpoint definitions*; the active *task identifier* from the TASKS registry; *model* template identifiers and hyperparameters; the MLflow *registry* connection and artifact-store credentials; and the *static context* feature list broadcast as a time-invariant channel (Section 8). Appendix B reproduces an excerpt of the trainer’s reference configuration showing the per-category feature sets and the full registered task roster.

## 12. Engineering Choices and Limitations

The proposed framework is underpinned by several architectural and methodological design decisions that shape its interoperability, configurability, explainability, and extensibility. This section discusses the rationale behind these choices, their implications for the software architecture, and the current limitations and future development of the platform.

### 12.1. Representation and Harmonization

A unified long-format event log is the single intermediate representation, so every new source needs only a loader and adapter, and no downstream component depends on FHIR or the non-FHIR API. It resolves three kinds of heterogeneity (Section 4 and Section 5): *source* (resource-specific extractors flatten every input to one nine-column spine, with a single patient-identifier normalization making all joins trivial), *semantic* (merged data dictionaries give each variable one meaning), and *temporal* (projection onto a common consent-relative day index with per-class aggregation yields a dense $\left[N_{\mathrm{pat}},T,F\right]$ tensor with an explicit missingness mask). Harmonization enables prediction but does not by itself establish the clinical value of the non-clinical streams, which is reported with the validation caveats below.

### 12.2. Consent-Relative Time and Seasonality

Expressing time on the consent-relative day index eliminates cohort calendar leakage but hides *absolute* seasonal signals (e.g., winter HF exacerbations) from the sequence axis. This is reversible: because the consent timestamp is retained, calendar features (month, season, or a day-of-year sine/cosine encoding) can be added as *static*, time-invariant covariates—broadcast across time steps for sequence models, or joined to the flat vector for trees (Section 8)—recovering seasonality without reintroducing wall-clock leakage. Whether this helps a given endpoint is directly testable from the configuration.

### 12.3. Mask Channel and Imputation Bias

An explicit mask alongside imputation is the pragmatic choice for sparse data. The mask does not by itself remove the risk that a poorly chosen per-variable imputation policy biases inputs, but three mechanisms bound and expose that risk: the mask lets the model discount imputed values; the default policy is conservative (linear interpolation only for short gaps in continuous variables, forward-fill for categoricals, with physiological range checks); and, because the imputation method is one of eleven per-variable configuration choices, the sensitivity of any result to it is a reproducible experiment. We therefore do not claim the default is unbiased everywhere, only that residual bias is bounded and explicitly testable.

### 12.4. Attributions and Their Clinical Validation

SHAP (marginal contribution under a background), integrated gradients, and temporal perturbation (sensitivity to ablation) provide complementary approaches for explaining model predictions and are integrated into a unified explainability framework within the proposed architecture. The current work demonstrates the technical implementation of these components and their integration into clinician-oriented narratives. Although the explainability framework was developed in collaboration with cardiologists, the clinical usefulness of these explanations—including clinician agreement, interpretability, usability, and their impact on clinical decision-making—has not yet been evaluated. These aspects require dedicated qualitative prospective studies with clinician participation and therefore fall outside the scope of the present architecture paper.

### 12.5. Evaluation Framework

The architecture includes a configurable evaluation module supporting patient-level data partitioning, standardized prediction task definitions, multiple machine learning model families, and reproducible experiment tracking. Together with the consent-relative temporal representation, missingness-aware preprocessing, and static-context integration, this provides a unified environment for developing and evaluating multimodal clinical prediction models.

### 12.6. Transfer Learning for Underpowered Endpoints

Several endpoints, notably HT rejection and LVAD-related infection, are limited by their small per-category positive counts (Section 3) and cannot be reliably evaluated on the present cohort. For these underpowered endpoints, transfer learning is the most immediate mitigation short of a larger cohort: the better-powered signals (dense wearable streams and HF admission) can pretrain a representation that is then fine-tuned on the scarce endpoints such as HT rejection or driveline infection [36], which the shared representation and task registry make straightforward. This complements the federated direction below, which instead pools data.

The RETENTION cohort is relatively small (390 patients) and was recruited from a limited number of clinical sites. Rare outcomes, including HT rejection and LVAD-related infection, are therefore infrequent, limiting the extent to which all prediction tasks supported by the framework can be evaluated within the present dataset. The cohort may also not fully represent the broader populations in which the framework could ultimately be applied. More generally, the performance of models developed using the framework depends on the quality, completeness, and representativeness of the underlying data, and upstream data-quality limitations may propagate through the analytical pipeline. In addition, several outcome labels in the present demonstration are proxy-derived and unadjudicated, most notably the use of unscheduled encounters as a surrogate for HF-related admissions, and should not be interpreted as adjudicated clinical outcomes until the planned clinician-led adjudication is complete.

Although the framework supports multiple ML model families through a unified architecture, the availability of numerous configurable model templates carries overfitting risk on a small cohort of this size. Furthermore, interoperability with additional EHR vendors, non-FHIR systems, and other digital health infrastructures has not yet been demonstrated. Finally, the computational requirements associated with sequence models and the explainability pipeline may limit deployment in resource-constrained environments, while the LLM-generated case reports currently rely on clinician verification and have not yet undergone automated faithfulness evaluation. From a clinical perspective, routine deployment will require integration with hospital information systems and AI governance processes beyond the trial-specific research environment, sustainable multimodal data acquisition, mitigation of alert fatigue through appropriate clinical workflows, and compliance with applicable medical-device regulations and data-protection requirements. Accordingly, the proposed framework should currently be regarded as a research and engineering platform that provides the infrastructure for future clinical AI applications and does not constitute evidence of clinical readiness.

### 12.7. Future Directions

The limitations map onto concrete next steps: multicenter external validation (beyond leave-one-site-out, ideally outside RETENTION); prospective rather than retrospective evaluation to establish clinical utility; real-time streaming deployment; tighter EHR integration; federated learning across sites to enlarge and diversify training without centralizing data; systematic fairness and bias assessment across groups, disease categories, and sites; and richer LLM-based conversational explanations grounded in the same feature attributions (Section 9).

## 13. Summary

The pipeline is a faithful realization of the five-stage schema in Figure 2. Streams are aligned into a unified event log; the signal is reconstructed through completeness filtering and configurable imputation with an explicit mask channel; prediction targets are quantified through the task abstraction; adverse events are predicted by a zoo of tabular and temporal models and explained through SHAP and temporal-perturbation attributions translated into clinical narratives. The whole system is declaratively configured, MLflow-tracked, and accessible both from a command-line interface and from an interactive web UI.

This work presents a modular, end-to-end software framework for multimodal clinical time-series prediction. The proposed architecture integrates heterogeneous HL7 FHIR and non-FHIR data sources into a unified longitudinal representation, supports configurable data harmonization, temporal processing, prediction-task definition, explainability, and reproducible model development within a single platform. Its modular, interoperable, and disease-agnostic design provides a scalable foundation for developing and deploying explainable clinical prediction systems across diverse healthcare settings and longitudinal clinical applications.

## 14. Availability and Reproducibility

The full source code of the pipeline, including data ingestion and harmonization, the unified event log and panel construction, the model zoo, the explainability stack, and the command-line interface, is released as open source under the Apache 2.0 license and is publicly available on GitLab at https://gitlab.com/retention-eu/retention-ai/-/blob/main/src/models/pipeline/training.py, accessed on 10 July 2026, with the corresponding operator UI repository available at https://gitlab.com/retention-eu/retention-ai/-/blob/main/src/gic_interface/components/model_trainer.py?ref_type=heads, accessed on 10 July 2026. Reproducibility rests on four mechanisms described in the preceding sections. First, a single declarative YAML configuration file (Section 11) captures every source path, cohort enumeration, sequence parameter, prediction-task definition, and model hyperparameter; its full schema, of which Listing A1 is an excerpt, ships with the repository together with a documented reference configuration. Second, MLflow [18] records hyperparameters, metrics, and artifacts for every run, so results are traceable to the exact configuration that produced them. Third, checkpoint persistence is versioned by task identifier and fold. Fourth, the CLI and the operator UI share the same task registry and configuration contract, so any run launched from the interface can be reproduced from the command line, and vice versa, using an identical configuration file. The FHIR ingestion layer targets any conformant HL7 FHIR R4 server that exposes an OAuth2 token endpoint; the RETENTION deployment is one such configuration, and the server base URL, authentication realm, and non-FHIR endpoint are all supplied through configuration and are not hard-coded into the application. No patient-level data are released; the RETENTION cohort is governed by its consent and data-use agreements.

## Figures and Tables

**Table 1:** Capability comparison of the proposed framework with representative end-to-end clinical AI systems. ✔ = supported; ∘ = partially supported or supported for a restricted case; ✘ = not supported. Assessment reflects the published descriptions and public documentation of each system as of August 2026; capabilities are compared with respect to the requirements set out in Section 1, and their absence in a given system reflects a different design scope rather than a deficiency.

Capability	Rajkomar et al. [9]	HAIM [10]	Clairvoyance [11]	PyHealth [12]	This Work
*Data integration*					
Standardized FHIR resources	✔	✘	✘	✔ ^a^	✔
Ingestion from a live authenticated endpoint	✘	✘	✘	✘	✔
Non-FHIR remote-monitoring/wearable streams fused with clinical data	✘	✘	✘	∘ ^b^	✔
Questionnaires/patient-reported outcomes	✘	∘ ^c^	✘	✘	✔
Unified longitudinal event representation	✔	∘	✔	✔	✔
*Temporal representation*					
Leakage-aware relative time axis	∘	∘	∘	∘	✔
Configurable temporal aggregation	✘	✘	✔	∘	✔
Configurable per-variable imputation	✘	✘	✔	∘	✔
Explicit observed-vs-imputed mask channel	✘	✘	∘	∘	✔
*Modeling*					
Declarative prediction-task abstraction	✘	∘	✔	✔	✔
Tabular *and* sequence model families	∘	∘	✔	✔	✔
Time-to-event and count endpoints	✘	✘	✔	∘	✔
Patient-level partitioning enforced by construction	✔	✔	✔	✔	✔
*Explainability*					
Feature or modality attribution	∘ ^d^	✔	✔	∘	✔
Temporal attribution	∘	✘	∘	∘	✔
Clinician-facing narrative generation	✘	✘ ^e^	✘	✘	✔
*Deployment*					
Experiment tracking and model registry	✘	✘	✘	∘	✔
Declarative single-file configuration	✘	✘	∘	∘	✔
Non-programmer operator interface	✘	✘	✘	✘	✔
Open-source release	✘	✔	✔	✔	✔
*Demonstration setting*					
Population	Inpatient EHR	ICU (MIMIC-IV)	ICU, wards, outpatient	ICU and public corpora	Advanced HF, LVAD, HT
Data provenance	Retrospective, 2 sites	Retrospective	Retrospective	Retrospective	Prospective, 6 sites

^a^ PyHealth 2.0 provides a MIMIC-IV-on-FHIR loader and a generic YAML-configured loader for NDJSON FHIR exports; ingestion is from static files, not from a live FHIR server. ^b^ PyHealth includes standalone wearable and physiological-signal corpora as separate datasets, but does not join patient-generated monitoring streams to EHR resources within one longitudinal patient representation. ^c^ HAIM ingests unstructured clinical notes as a text modality, but not structured patient-reported questionnaire instruments. ^d^ Attribution is provided through attention weights over the input timeline without a dedicated attribution module. ^e^ A subsequent extension of HAIM couples the framework with generative models to produce explanatory summaries; the comparison here refers to the originally published framework.

**Table 2:** The unified event-log schema: one row per atomic clinical event. Representative rows from five sources illustrate how heterogeneous inputs are unified behind a common nine-column spine. The day index is days since the patient’s consent date, used as the model’s leakage-aware time axis.

	Patient_id	Timestamp	Source	Variable	Value	Unit	Label	Extra	Dayindex
*obs*	P0042	2024-03-12	observation	Systolic BP	138	mmHg	vital-signs	{…}	21
*enc*	P0042	2024-04-03	encounter	Ambulatory	AMB	–	Encounter	finished	43
*q*	P0042	2024-04-10	questionnaire	Dyspnea at rest	1.0	–	QSymptoms	yes	50
*dev*	P0042	2024-04-11	nonfhir_meas	Weight	82.4	kg	Weight	scale-01	51
*med*	P0042	2024-04-11	medication	Furosemide	1.0	–	MedStatement	active	51

Row labels denote the event source: obs, FHIR Observation; enc, FHIR Encounter; q, questionnaire response; dev, non-FHIR device measurement; med, medication statement. {…} indicates content omitted for brevity.

**Table 3:** Prediction tasks currently registered in the system. All tasks share the same cohort-filter and label-builder plumbing and are swappable from configuration.

Kind	Outcome	Cohort
Binary, within *H* days	Mortality (any)	All
Binary, within *H* days	HF admission	HF/VAD
Binary, within *H* days	HF decompensation	HF/VAD
Binary, within *H* days	CV admission	All
Binary, within *H* days	HT rejection	HT
Binary, within *H* days	Driveline infection	LVAD
Time-to-event regression	Days to next HF admission	HF/VAD
Count-within	HF encounter count in horizon	HF/VAD
Multi-class	Evolution (better/stable/worse)	All
Regression	Length of stay	All

## Data Availability

The source code of the framework is available on GitLab, as described in Section 14. Patient-level data from the RETENTION trial are not publicly available because of privacy restrictions and the trial’s informed-consent and data-use agreements.

## Appendix A. Operator Interface: Page-by-Page Detail

This appendix expands Section 10 with the tab-level layout of each of the four operator-interface pages.

### Appendix A.1. Session and Navigation

On first load, the user provides a name and selects a role (Researcher, Data Analyst, Clinician, Administrator); the choice is held in the session state and is used to scope subsequent interactions. The sidebar then exposes a card-style navigation menu with four entries (Home, Configuration, Model Trainer, and Patient Completeness) and a logout action that clears the session. The home dashboard is intentionally light: it summarizes what each of the other three pages does and acts as a landing point once the user is authenticated.

### Appendix A.2. Configuration Page

The configuration page is an in-browser editor for the pipeline’s single YAML configuration file. The file is uploaded via the action bar at the top, parsed into a typed Config object, and materialized in the session state along with a derived data frame read from the path it points to. Two indicators confirm the load: a file name caption and a row/column count for the wide matrix. A toggle switches between *view mode* and *edit mode*, and a modified flag tracks whether unsaved changes exist.

The configuration is then split across five tabs, whose semantics map one-to-one onto the configuration sections described in Section 11:

- **Data Paths.** Per-source CSV paths with live existence checks. Each row shows the key, the path, and a tick or cross indicator; aggregate counters at the top of the tab summarize how many of the configured paths resolve on disk. New paths can be added inline.
- **Study Settings.** Four dictionary editors for clinical sites, treatment groups, patient categories, and recruitment status. The editors enforce that identifiers stay stable while values are renamed.
- **Sequence & Evolution.** Typed editors for the sequence parameters (horizon, window, step, aggregation, minimum coverage) and for the evolution target configuration (variable, lookahead, delta threshold, direction). Each field uses the appropriate widget kind (checkbox, number, or constrained drop-down), which removes a class of typing errors that text-only YAML editing would allow.
- **Advanced.** Task identifier, MLflow registry connection, and the static context feature set. Secret-like keys are masked.
- **Raw YAML.** A textarea fallback with explicit *Validate & Apply* that re-parses the document before letting the user save it, plus a download button to export the modified configuration.

### Appendix A.3. Model Trainer Page

The trainer page is the centerpiece of the GIC-side workflow. It is organized in four tabs:

- **Setup & Train.** The user picks a prediction task from the registered roster (the same TASKS registry used by the CLI), reads its objective, label kind, and lookahead in an inline expander, and then chooses a training configuration. Trainer hyperparameters (epochs, batch size, learning rate, hidden dim, LSTM layer count, dropout, bidirectionality, early stopping patience, weighted loss toggle) are exposed as sliders and select sliders. A device selector resolves *Auto*/*CPU*/*GPU* against the local CUDA availability and falls back to CPU with a warning when a GPU is requested but unavailable. Pipeline level settings cover sequence length, train/eval ratios, random seed, the number of explainability samples, IG steps and SHAP background size, and an experiment name. The MLflow connection block accepts the tracking URI, credentials, and the matching S3 endpoint and access keys for the artifact store; both URL fields are validated against an HTTP(S) regex before any environment variable is set. Pressing *Start Training* dispatches run_pipeline in-process and wires a status callback into the page.
- **Live status.** The callback drives a progress bar, a one-line headline, and a per-epoch detail line that shows train/validation loss and any task-specific metrics. Pipeline events (data load, sequence build, split, label distribution build, training start, per-epoch ticks, training done, explainability, completion) are appended to a rolling log expander. A live label distribution table is updated as labels are built per split and per category, which lets the user catch class imbalance before training finishes.
- **Results.** For each completed run, the page renders the train/validation loss curve, the per-category evaluation metrics returned by the trainer (presented as metric tiles), and a placeholder for regression scatter plots that lives in the MLflow artifacts of the run.
- **Explainability.** Per task and per category, the page exposes five tabs that visualize the artifacts produced by the explainability stack of Section 9: mean |IG| feature importance, mean |SHAP| feature importance, the SHAP summary plot, mean attention weights across timesteps, and the timestep importance curve. A slider controls the top-*N* feature cutoff shared by the two importance tabs.
- **MLflow.** The fourth tab surfaces the run identifier and a deep link into the MLflow UI for each completed task, so that the user can inspect the full run, download artifacts, and assign a registry alias.

### Appendix A.4. Patient Completeness Page

The completeness dashboard is the diagnostic counterpart to the trainer. On entry, it builds a PatientCompleteness engine backed by a PatientEvolutionPipeline; the events-long table, patients table, observations, and encounters are loaded once and cached against a hash of the active YAML configuration so that reruns on the same configuration are instant. The page then exposes four tabs:

- **Baseline overview.** Computes per-site and per-patient baseline observation summaries, renders two ranked bar charts (total events per site and maximum per-patient events per site), and lets the user drill down into a single patient’s per-variable baseline counts.
- **Milestone analysis.** A milestone selector covering baseline, the regular four-month milestones, and the unscheduled bucket. The user can compute either variable counts or completeness for the selected milestone; results are presented as overall and per-site tables along with a stack of per-site Altair charts.
- **Events per patient.** Paired site/source filter with a global view (events by source per patient) and a per-variable view (events broken down by variable, with summary metrics, a top-15 chart, and a full table in an expander).
- **Observation/encounter correlation.** Two association modes: by day index and by same calendar day. Each produces summary metric tiles for each encounter category (scheduled, unscheduled, scheduled in-person, unscheduled in-person, uncharted), bar plus donut charts for the category mix, a top-30 stacked bar breakdown per patient, and detail tables in collapsed expanders.

## Appendix B. Configuration Schema Example

Listing A1 expands Section 11 with an excerpt of the trainer’s reference configuration: the per-category feature sets and the full roster of registered prediction tasks.

**Listing A1:** Per category feature sets and prediction task definitions consumed by the trainer. Each task declares its source label column(s), the integer values that count as positive, the feature horizon in days, and the class mapping; the same registry is exposed by the Model Trainer page.
